# Efficient Active Sensing with Categorized Further Explorations for a Home Behavior-Monitoring Robot

**DOI:** 10.1155/2017/6952695

**Published:** 2017-11-28

**Authors:** Wenwei Yu, Keigo Nakahata, Guang Hao Sun, Akio Namiki, Sayuri Suwa, Mayuko Tsujimura, Le Xie, Jinwu Wang, Shao Ying Huang

**Affiliations:** ^1^Center for Frontier Medical Engineering, Chiba University, Chiba, Japan; ^2^Graduate School of Engineering, Chiba University, Chiba, Japan; ^3^Graduate School of Informatics and Engineering, The University of Electro-Communications, Chofu, Japan; ^4^Graduate School of Nursing, Chiba University, Chiba, Japan; ^5^Research Institute of Med-X, Shanghai Jiao Tong University, Shanghai, China; ^6^Engineering Product Development, Singapore University of Technology and Design, Singapore

## Abstract

Mobile robotics is a potential solution to home behavior monitoring for the elderly. For a mobile robot in the real world, there are several types of uncertainties for its perceptions, such as the ambiguity between a target object and the surrounding objects and occlusions by furniture. The problem could be more serious for a home behavior-monitoring system, which aims to accurately recognize the activity of a target person, in spite of these uncertainties. It detects irregularities and categorizes situations requiring further explorations, which strategically maximize the information needed for activity recognition while minimizing the costs. Two schemes of active sensing, based on two irregularity detections, namely, heuristic-based and template-matching-based irregularity detections, were implemented and examined for body contour-based activity recognition. Their time cost and accuracy in activity recognition were evaluated through experiments in both a controlled scenario and a home living scenario. Experiment results showed that the categorized further explorations guided the robot system to sense the target person actively. As a result, with the proposed approach, the robot system has achieved higher accuracy of activity recognition.

## 1. Introduction

Increasing population age turns to be a significantly serious problem in the world [1, 2]. As the population of the single-living elderly (SLE) is increasing rapidly, the demand for behavior monitoring of SLE at home is rising. This is due to the necessity to secure their safety, to know the rhythm and quality of their ADL (activity of daily living), and to make effective care plans.

In the literature, two main approaches have been reported on elderly behavior monitoring at home: wearable systems and smart houses [3]. Wearable systems may cause user discomfort which leads to discontinuous monitoring [4]. Smart houses usually require a large number of sensors which increases the cost and the operational complexity. Even more, blind spots and dead angle due to the furniture layout may interrupt the monitoring, which threatens the safety of the person under monitoring. Recent studies have shown that robots can be an important tool for facilitating part of ADL, exercise, and rehabilitation in a home environment through interactions with the elderly [5–8].

Our studies showed that mobile robotics is a potential solution to home behavior monitoring for the elderly [9–11]. A replacement of smart house with a mobile robot reduces the number of sensors which in turn reduces the cost of implementation and the deployment complexity. Moreover and importantly, it provides seamless temporal and spatial monitoring for safe home daily living if the robot is well controlled. It was reported in [9–11] that a mobile robot was capable of tracking a target subject and performing tasks such as observations and analyses of the environment and recognition and analyses of the behaviors of the subject, by using the location information and the extracted body contour features of the subject. The system showed an excellent performance: 98.6–99.4% as an overall correct rate of human activity recognition in testing datasets. However, the test data sets were collected in a static scenario where the activities were carried out sequentially at one place and repeated during a certain period of time.

In a real home environment, there are various types of uncertainties, such as the ambiguity between the subject under monitoring and the surrounding objects, the occlusion by furniture, difficulties in localization and movement control due to uneven floor, and frequent partial furniture layout alternations. In this study, we focused on the ambiguity between the subject under monitoring and the surrounding objects, which is a major obstacle for home behavior recognition. Active sensing is the ability to infer information under an uncertain environment by spontaneous sensing activity [12]. There are mainly two categories. The first category is the use of self-generated signals to probe the environment, such as echolocation chirps in bats. The other one uses a self-motion, such as a move to find out an object hidden in the shadows [12]. The active sensing mechanism in this study was inspired by the latter. There are various strategies for active sensing for a robot in terms of maximizing the information gain and minimizing the cost simultaneously [13–16]. In this work, we propose a strategy of active sensing that suits the aforementioned home monitoring scenario, which identifies the situation and conducts a categorization of the situations before further explorations. It is named active sensing with categorized further explorations. For identifying different situations in an uncertain scenario, the heuristic-based and the template-matching-based irregularity detections were implemented and compared. Their time cost and accuracy in activity recognition were evaluated in different uncertain situations.

This paper is organized as follows.  Section 2 describes the active sensing framework briefly and the proposed strategy.  Section 3 details the experiments to test the different irregularity detections for the categorization for the proposed approach. In Section 4, experimental results are presented, which is followed by discussions. Concluding remarks are stated in Section 5.

## 2. Methods

In this section, we present the details of the proposed active sensing with categorized further exploration for a mobile robot for home behavior monitoring, including the framework and the categorization. For the categorization, the heuristic-based and the template-matching-based methods are examined and compared in terms of time cost and recognition accuracy.

### 2.1. The Mobile Behavior-Monitoring Robot

**Figure 1:**
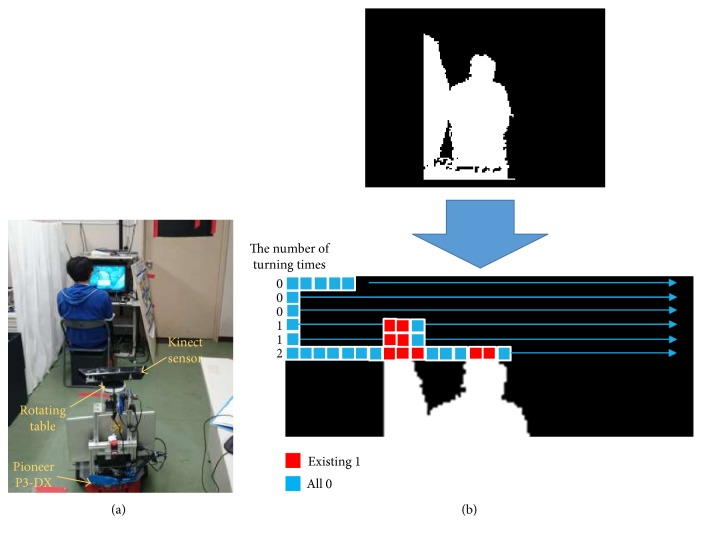
How to judge the irregularity of a feature extraction. (a) The scenario where there is an ambiguity between the target subject and the surrounding object. (b) A binary image in the human body contour region used for activity recognition.

The autonomous mobile behavior-monitoring robot performs the subject tracking and activity recognition. As shown in Figure 1(a), the hardware of the robot is assembled base on Pioneer P3-DX. It includes a laser range finder (LRF) and a Kinect sensor on a rotating platform. The LRF provides data about the obstacles in the environment, and it is used to determine the location of the robot and to avoid obstacles. The Kinect sensor is used to detect and track the subject. This sensor is mounted on a rotating platform. In the robot, an algorithm is applied to integrate Kinect and Lidar (light detection and ranging) sensor data. It is for detecting and tracking novelties using the environment map of the robot as a top-down approach without the necessity of large amount of training data. Using geometric features calculated from human body contour extracted from depth images, the system can identify six different activities: standing, walking, bending, sitting, lying down, and falling [9–11].

### 2.2. A Framework of Active Sensing for Home Monitoring Mobile Robots

Active sensing is the ability to infer information under an uncertain environment by spontaneous sensing activities [12–17]. There are two types of active sensing, one uses self-generated signals to probe the environment, such as echolocation chirps in bats [16], and the other uses a self-motion, such as a move to find out an object hidden in the shadows [12]. The active sensing mechanism implemented in the mobile behavior-monitoring robot is inspired by the second type.

In the literature, the active sensing has been studied as a theoretic framework for understanding biological sensing mechanism [15–17]. One control framework for active sensing by the human sight is Context-Dependent Active Controller (C-DAC). C-DAC assumes that the observer aims to optimize a context-sensitive objective function that takes into account behavioral costs such as temporal delay, response error, and the cost of switching from one sensing location to another [17]. This framework allows us to derive behaviorally optimal procedures for making decisions about where to acquire sensory inputs, when to move from one observation location to another, and how to negotiate the exploration-exploitation tradeoff between collecting additional data versus terminating the observation process [17].

The implementation to the mobile robotics focused on the efficient information collection, or exploration of unknown areas [12, 13], and the locations of a team of mobile robots for efficient informative measurement [14]. In the case of home behavior monitoring, the routine tasks for the mobile robot are target person following, visual tracking, and behavior recognition. Especially, the former two tasks need reactive planning for ensuring the safety of the target person, not to obstruct the path of the target person, while not losing him/her from its visual field. Thus, it is important to identify the situations that require further explorations and determine how important it is to deal with the situation. Our categorizing and further exploration strategy for active sensing fits well to the application for high detection accuracy and time cost minimization simultaneously.

For a home monitoring robot, in the case that a subject sits down close to an object, there is ambiguity between the targeted subject and the surrounding object. In the algorithm, the subject and the object are at the same depth (Figure 1(a)) where the human body contour extraction fails and part of the object is included in the subject contour. Therefore, active sensing is necessary to deal with such cases. In order to implement both the situation categorization and active sensing, three algorithms were performed in a row: (1) categorization by detecting irregularity of feature extractions, (2) further explorations by adjusting sensing parameters of the robot accordingly, and (3) avoiding excessive parameter adjustment based on a short-term memory mechanism so that the behavioral cost (the time of changing the parameter) was minimized. In the proposed approach, the first step is to identify when this situation is occurring. In this paper, both heuristic-based and template-matching-based active sensing schemes are applied for irregularity detections. They are examined and compared through experiments.

### 2.3. Active Sensing with Irregularity Detections

In the proposed framework, active sensing with categorized further explorations, irregularity detection is a key step for categorization of the situation and further explorations. Irregularity detections (also known as anomaly detection) which is to identify different situations in an uncertain scenario for home monitoring for the elderly are conducted using active sensing. Two types of irregularity detections were implemented and compared. One is heuristic-based irregularity detection and the other is template-matching-based irregularity detection. The heuristic-based active sensing (H-AS) is a method specifically designed for the targeted application. The template-matching-based active sensing (TM-AS) is a clustering-based irregularity detection which is one of the machine learning-based approaches.

#### 2.3.1. Heuristic-Based Active Sensing (H-AS)

H-AS is specifically designed to detect the situation where the subject under monitoring is indistinguishable with the surrounding (e.g., a wall or a curtain) for home monitoring, which is the irregularity for detection.

Figure 1(a) shows the scenario when the robot is facing towards the subject under monitoring. The robot acquires images of the subject. The image acquired is scanned in blocks of 5 × 5 pixels with an aim of detecting more than one block per line where the pixels take a value of zero. When the image has one or none of this blocks per line, it is considered as a suitable image for the body contour extraction and there is no need for the robot to conduct active sensing at that location.

The human body contour region which is used for activity recognition is expressed in a binary image as shown in Figure 1(b). In the situation illustrated on the line at the bottom in the binary image in Figure 1(b), the human body contour becomes indistinguishable with part of the wall located at the left side of the subject. In this scenario, a correct feature extraction is not possible and the robot fails in activity recognition. This is considered as an irregularity. Further explorations are needed at this location.

Once an irregular situation is identified, a straightforward next step is to move the robot to a new location, where it will have a different viewpoint of the scene. In this study, in order to optimize the active sensing operation time, instead of moving the robot to a different location, rotational movements by rotating the rotating platform (refer to Figure 1(a)) are implemented. The rotation is done in both directions until it can identify a regular situation in the body extraction process [18].

#### 2.3.2. Template-Matching-Based Active Sensing (TM-AS)

The final technical goal of this study is to enable the robot to recognize robustly a number of behaviors aforementioned (standing, walking, bending, sitting, lying down, and falling). TM-AS is applied. For these behaviors, when the standard patterns of all the behaviors can be collected and a regular pattern space can be expanded, then this space can be used to check an arbitrary pattern to see whether it is regular or irregular. TM-AS is a clustering-based anomaly detection where machine learning is applied. Certainly, the discriminability depends on the patterns collected, features of the patterns, and discriminability indexes employed. These factors were investigated through experiments. The study will be reported in the following sessions. Note that, though the method is implemented for a specific depth sensing, the proposed idea can be applied to the other sensing modals in general.

*(1) Template Preparation*. Since, confusing with the environment is less possible to occur for the behaviors of walking and falling down, in this study, the templates would be at first setup for the other four behaviors (standing, sitting, lying down, and bending) which are more possible to occur near the objects (such as wall, furniture, and curtain) in the living environment.

**Figure 2:**
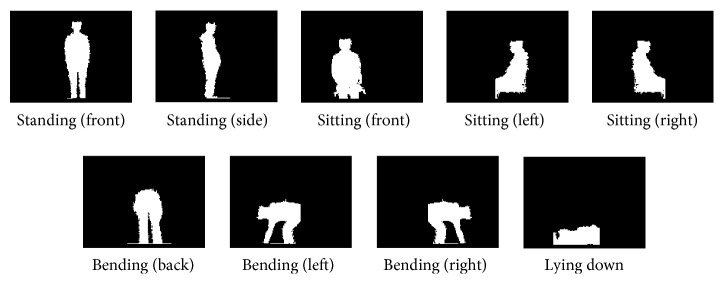
Images as templates for different activities.

Standard depth images were recorded for each behavior as follows: (1) the depth images were collected 1.3 m away from the targeted person. (2) The numbers of templates for each different behavior were set as 2 for standing, 3 for sitting, 3 for bending, and 1 for lying down. If the patterns taken from the left and those from the right are different sufficiently, they were treated as different templates.  Figure 2 shows the template patterns.

*(2) Feature Selection and Similarity Indexes*. Since it is easier to extract contour of depth images, three contour-related parameters were considered. They are the distance between the center of weight to the contour point (*D*(*i*)), and the angle of normal vector (*A*_nv_(*i*)) as shown in (1), (2), and (3), respectively.

(1) $$\begin{array}{c} D\left(i\right)=\sqrt{{\left(x_{i}-x_{c}\right)}^{2}+{\left(y_{i}-y_{c}\right)}^{2},} \end{array}$$

where (*x*_*c*_, *y*_*c*_) denotes the coordinate of the center of weight, and (*x*_*i*_, *y*_*i*_) denotes the *i*th contour point.

(2) $$\begin{array}{c} A_{tl}\left(i\right)=\tan^{-1}\left(\frac{y_{i+P}-y_{i-P}}{x_{i+P}-x_{i-P}}\right), \end{array}$$

(3) $$\begin{array}{c} A_{nv}\left(i\right)=A_{tl}\left(i\right)-\left(\frac{\pi }{2}\right), \end{array}$$

where the subscript tl stands for tangential line of the contour points, and nv stands for the normal vector. The accumulated difference (AD) between a template image and the one under test is expressed as

(4) $$\begin{array}{c} {AD}_{D}=\sum_{i=1-n}\left|D_{I1}\left(i\right)-D_{I2}\left(i\right)\right|,\\ {AD}_{Anv}=\sum_{i=1-n}\left|A_{nv\_I1}\left(i\right)-A_{nv\_I2}\left(i\right)\right|, \end{array}$$

where AD_*d*_^upper^ and AD_*d*_^lower^ are the accumulated difference of distance of contour points for the upper and the lower body, respectively. Points that have a difference with the top pixel along the longitudinal direction smaller than a threshold are defined as upper body contour points and the others are the lower body ones. AD_Anv_ is the accumulated difference of angle in radian of the unit principal normal vector.

The upper body feature, AD_*d*_^upper^, can make a difference between the class of standing and sitting and the other class including bending and lying down. The lower body feature, AD_*d*_^lower^, can tell the difference among the four activities. On the other hand, the angle of unit normal vector (AD_Anv_) might be sensitive to local changes. Both of the accumulated difference in distance and angle of unit normal vector will be affected by occlusion and cluttering. In this study, either the accumulated difference in distance or angle is determined as the similarity index for template matching, by a score calculated from the template patterns. In the future, they can be combined to deal with different uncertainties.

Davies-Bouldin (DB) index is a clustering index which is a ratio of intraclass distance and interclass distance. A small DB index indicates intraclass similarity and interclass difference. It is used to evaluate whether the accumulated difference of distance or that of angles is more informative for classification for TM-AS. A smaller intraclass or a larger interclass distance results in a smaller index value. That is, a feature leading to a smaller DB index value shall be a better feature.

(5) $$\begin{array}{c} DB=\frac{1}{k}\sum_{i=1-k}\max_{j\neq i}\left(D_{ij}\right),\\ D_{ij}=\frac{d_{i}+d_{j}}{d_{ij}}. \end{array}$$

### 2.4. Modification of Sensing Parameters

Two sensing parameters can be modified by the system.

**Figure 3:**
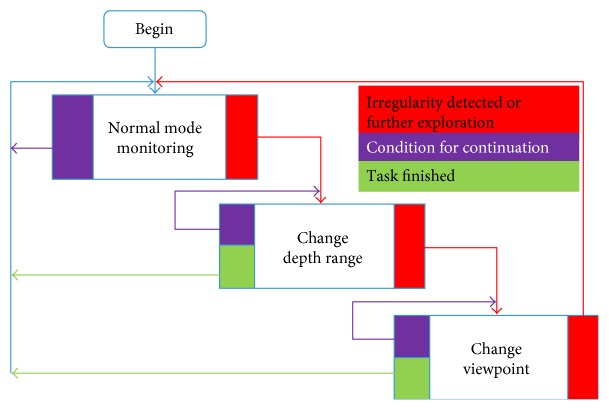
The configuration of the robot and the subject under test in experiment 1.

1. The viewpoint: the angular relative location of the robot with respect to the targeted subject as shown in one of the experimental setups shown in Figure 3. In this study, the robot rotates the sensor to change its viewpoint until the irregularity disappears.
2. The range of depth: the probabilistic depth map estimation [12] could be expressed by (6) and (7). *D*_*xy*_ is the raw image value from the depth camera at the coordinates *x* and *y*. *I*_*xy*_ is the extracted image value determined by the probability Prob_*xy*_ calculated by (6), using a threshold Th_Prob_. This can be interpreted as the robot observes the target at a gaze distance *μ*, with range of depth *σ*.

(6) $$\begin{array}{c} {Prob}_{xy}=\frac{1}{\sigma \sqrt{2\pi }}\exp\left[\frac{-{\left(D_{xy}-\mu \right)}^{2}}{2\sigma^{2}}\right] \end{array}$$

(7) $$\begin{array}{c} I_{xy}=\left\{\begin{array}{l} 1\;{Prob}_{xy}\geq {Th}_{Prob},\\ 0\;{Prob}_{xy}<{Th}_{Prob}. \end{array}\right. \end{array}$$

### 2.5. Irregularity Detections, Sensing Parameter Modification, and Behavior Recognition

**Figure 4:**
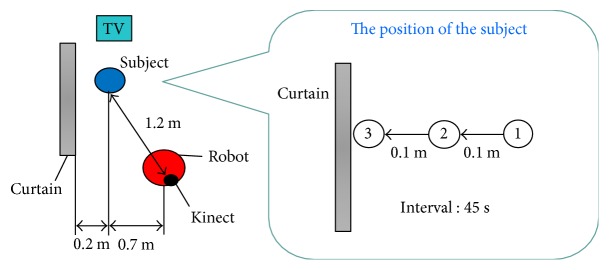
A flowchart of irregularity detection, sensing parameter explorations, and behavior recognitions.

Figure 4 shows a flowchart of irregularity detection, sensing parameter explorations, and behavior recognitions. Three modules, namely, normal mode monitoring, change depth range, and change viewpoint, were connected through three different types of links. The red path means irregularity detected or further explorations, the purple one shows condition for recursion, and the green one indicates the task finished and return.

In the normal mode monitoring, when a number of irregularities are detected, the system calls the module of change depth range. If the module finds a good depth range as a sensing parameter after exploration, it returns to the normal mode monitoring. Otherwise, the system shifts to change viewpoint module. Since the change of depth range does not need to change the physical position of the robot, its cost is lower than that of change viewpoint, which needs to physically change the orientation of the robot. The depth-first search strategy was employed. That is, for each viewpoint, a full span of depth range is explored. For the details of the modules, please see the appendix.

## 3. Experiments

This section describes the experiments for comparing and evaluating the heuristic-based and the template-matching-based active sensing schemes as well as the conventional approach without active sensing, in a controlled scenario and a daily life activity scenario. For TM-AS, the similarity index using DB index was evaluated based on the templates prepared, as shown in Section 2.3.2, item (1).

### 3.1. The Clustering Index (DB Index) Values for Evaluating AD_*d*_^upper^, AD_*d*_^lower^, and AD_Anv_ for TM-AS

For TM-AS, as presented in Section 2.3.2, item (1), the three features (AD_*d*_^upper^, AD_*d*_^lower^, and AD_Anv_) for calculating similarity indexes for template matching were evaluated by using the DB index. The one with the smallest DB index is selected as the similarity index.

### 3.2. Experiment 1, a Controlled Scenario

**Figure 5:**
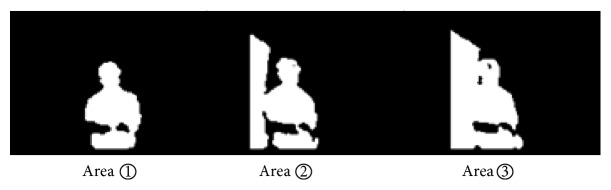
Observed and processed depth images in different areas.

The aim of this experiment is to evaluate the performance of the two active sensing schemes when the target person is sitting close to an object in the environment and indistinguishable in depth with the object. In this scenario, the subject is sitting beside a partition curtain, with a similar sitting height of the subject, as illustrated in Figure 1(a). The similar situations may be caused by the subject standing or sitting against the wall, or bending in front of a refrigerator with its door opened as a background. The robot was located (manually) at a position where the curtain interferes with the human body contour extraction as shown in Figure 3. Note, a similar situation would happen even if a different image feature extraction (e.g., the feature from color images) is employed. The subject was required to sit at one of the three locations with a distance of 0.1 m in between as shown in the insert in Figure 3.  Figure 5 shows the observed targeted person and the object in different areas.

In this scenario, we evaluated three different configurations, the conventional system (no active sensing), H-AS, and TM-AS. The evaluation includes not only the accuracy but also the number of rotations that it took for the robot to obtain the most appropriate recognition.

### 3.3. Experiment 2, Daily Living Activity Scenario 1

The aim of this experiment is to evaluate the active sensing performance in a flow of home living activities. In contrast with the previous experiment, the interference with surrounding objects does not necessarily happen for all the frames for all the activities. The experiment with a total duration of one hour was performed by two subjects for the conventional system (no active sensing) and by three subjects for the proposed active sensing scheme (due to the analysis on Experiment 1, only H-AS is implemented. For detailed reasons, please find them in Section 4.2). In this experiment, the robot tracks a subject moving in a home living environment. Therefore, the place where the robot stops is not always the same.

**Figure 6:**
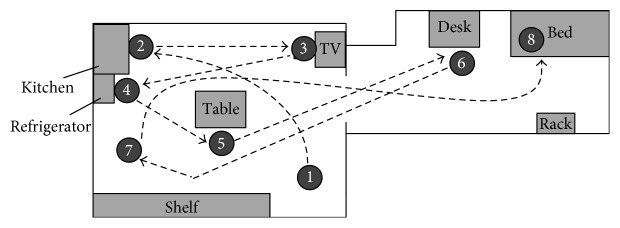
The layout of the two rooms for the planned situations and sites for experiment 2.

We set up two rooms as illustrated in Figure 6. The subject moved to follow a scenario performing the following activities: initially the subject arrived at home ❶. The robot was waiting at the entrance, and it started tracking the subject. Then, the subject moved towards the kitchen and washed his hands ❷. He walked to the TV, took a seat, and watched TV for a while ❸. After that, he stood up and picked a drink from the refrigerator ❹. When he finished his drink, he went to the table and read a newspaper for a while ❺. After that, he moved to his desk and read a book ❻. Some minutes later, the subject went to an open area and began to walk as an exercise ❼. When the exercise was finished, he went to bed ❽.

During the experiment, the robot tracked the subject and identified the performed activities (standing, walking, sitting, bending, and lying down) in real time and it also logged the information for further analysis. The full experiment was recorded with a video camera. Once the experiment was over, we analyzed the log file and the video data to calculate the accuracy of the recognition for each activity.

### 3.4. Experiment 3, Daily Living Activity Scenario 2

**Figure 7:**
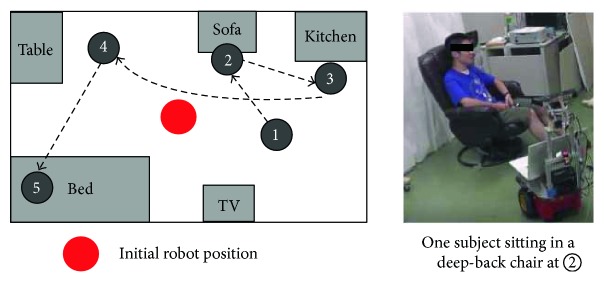
The layout of the one room for the planned situations and sites for experiment 3.

The aim of this experiment is different with that of experiment 2. Multiple confusing situations were contained in this scenario, as shown in Figure 7.

The subject moved from location ❶ to ❷ while the robot moved towards the subject to monitor his behavior from its initial position. The subject sat in a deep-back chair for the first five minutes, as shown in the left part of Figure 7. This deep-back chair was the source of the first confusion. Then the subject moved to ❸ and stood in the kitchen for cooking for the next five minutes. The subject then moved to ❹ and sat and had meal for another five minutes. In this situation, the wall was the source of confusion. Finally, the subject moved to the bed on the left lower corner at ❺ and lied on the bed for the final five minutes. Since the subject was asked to turn over on the bed, he was very close to the wall in some cases. This is the 3rd source of confusion. Both the H-AS and TM-AS were implemented and compared with the non-AS cases. Three subjects took part in the experiment.

## 4. Results

### 4.1. The Clustering Index for Different Features (AD_*d*_^upper^, AD_*d*_^lower^, and AD_Anv_)

DB index values for all the three features, AD_*d*_^upper^, AD_*d*_^lower^, and AD_Anv_, were calculated based on (1), (2), and (3) and are shown in Table 1. As noted in subsection 2.3.2, a smaller DB index value means better clustering, that is, a smaller intraclass distance and a larger interclass distance. As shown in Table 1, the accumulated difference of unit normal vector angle has the smallest value. Therefore, AD_Anv_ is used for further analyses next. Moreover, AD_*d*_^upper^ and AD_*d*_^lower^ are larger no matter it is upper body or lower body. It is observed that AD_*d*_^lower^ < AD_*d*_^upper^, which indicates that the contour of the lower body provides more useful information for measuring the similarity of images of different activities.

Table 2 shows the AD_Anv_ of each template image pair in Figure 2. Generally, the AD shows a smaller value for the images from the same class, for example, the standing (side) and standing (front), while those for the images from different classes, for example, standing and sitting, are larger.

According to this analysis, in experiments 1, 2, and 3, AD_Anv_ was used as the index for irregularity detection.

### 4.2. Experiment 1, a Controlled Scenario

The results of experiment 1 are summarized in Tables 3 and 4.  Table 3 tabulates the activity recognition accuracy for sitting position for the three areas. As shown in Table 3, for different distance between the subject and the curtain, the accuracy of activity recognition varies significantly for areas ② and ③ when the subject got closer to the curtain. As shown, without AS, it shows accurate detections for area ① but low accuracy for area ② and area ③. TM-AS shows the highest accuracy of activity recognition in all the three areas whereas H-AS achieves similar accuracy of activity recognition for areas ① and ② but cannot obtain high accuracy in ③.

**Figure 8:**
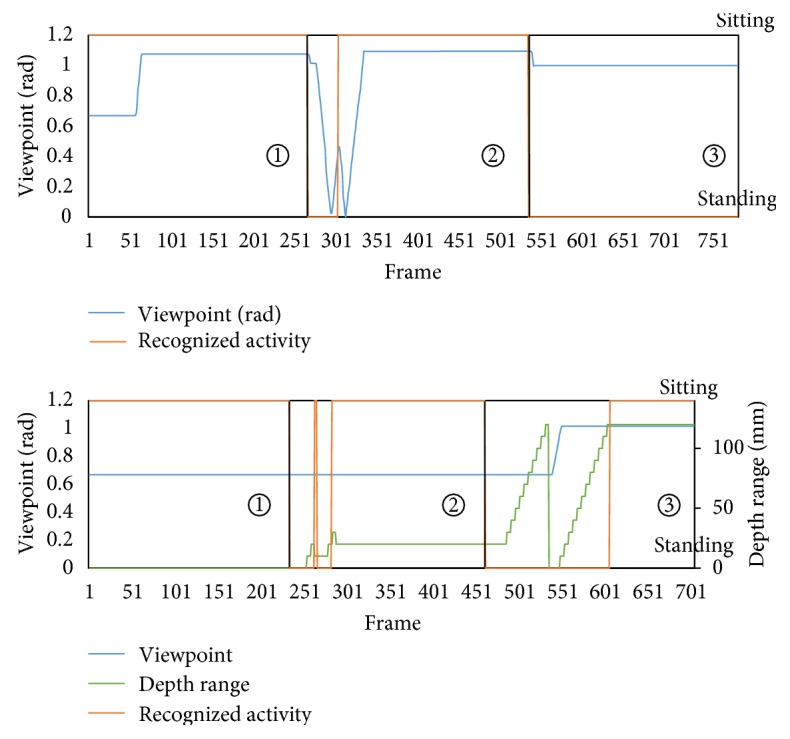
The sensing parameters and recognized activity of the two active sensing schemes. (a) H-AS; (b) TM-AS.

Figure 8 shows the change of the sensing parameters (viewpoint and depth range) and activity recognized (correct answer: sitting or incorrect answer: the others). Figure 8(a) shows the results for H-AS where only the viewpoint was changed whereas the lower graph shows those for TM-AS where both the depth range and viewpoint were changed. As shown in Figure 8(a), when the subject changed from area ① to ② getting closer to the curtain, the robot misrecognized the activity. With the heuristics, the irregularity was detected and the robot changed its viewpoint to obtain a correct recognition. But for area ③, H-AS failed the irregularity detections due to the limitation of the heuristics; thus, no change of viewpoints was conducted for a correct activity recognition.

**Figure 9:**
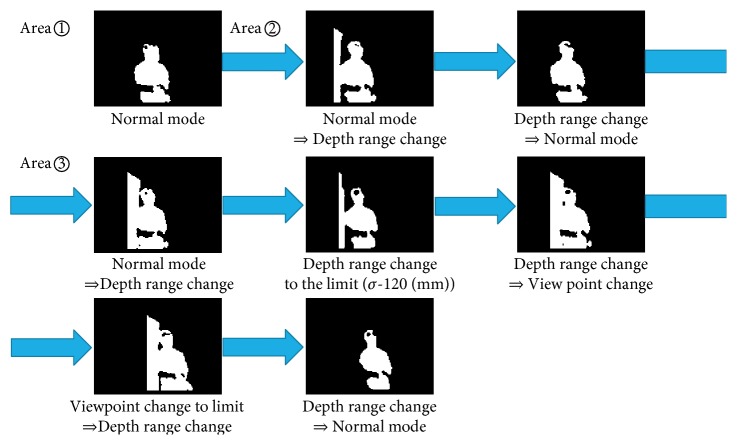
A sequence of selected image to show a TM-AS process as the subject moved.

The TM-AS is a more generalized irregularity detection function. In the experiment, it explored two sensing parameters: both viewpoint and depth range. This is because that without exploring both sensing parameters, it is impossible to deal with the ambiguous situations in area ③. Whenever the subject moved from area ① to ②, or from area ② to ③, the irregularity could be detected, and the exploration was initiated. After the subject moved from ① to ②, only changing the depth range was needed, and then the right activity was recognized. However, when the subject moved from ② to ③, the robot had to explore both the viewpoint and depth range to find the right sensing parameter for activity recognition. The detection process is further illustrated using depth images in Figure 9. In Figure 9, several selected observed images of the trial with TM-AS as the subject moved are shown. The confusion with the surrounding objects was reduced and finally disappeared, as multiple sensing parameters were explored.

Table 4 shows the time cost of both active sensing schemes in the areas ② and ③ for three trials. Although depending on small difference between the initial position, orientation, and the noise of depth images in some frames, the exploration process was different with different trials. Generally, the time cost of TM-AS is higher than that of H-AS in area ②. Here, the time cost was calculated by counting the time from the first irregularity detected to the change of the last sensing parameter. For TM-AS, the time cost in area ③ was higher than that in area ②.

Since the time efficiency is very important for real-time applications, and in the real daily living environment, the situation similar to that in area ③ would not frequently happen; thus in experiment 2, H-AS was further examined.

### 4.3. Experiment 2, Daily Living Activity Scenario 1

Table 5 shows the results of one subject in non-AS experiment. The walking in italic means the move from one site to another site. At ⑦ for walking exercise, since the subject was instructed to repeatedly move forward, stand, move backward, and stand slowly, two behaviors: standing (⑦-1) and walking (⑦-2), both in bold, shall be recognized. Here, we omitted transitional states, which are the short behaviors detected between two main behaviors, for example, between sitting at ③ and walking for ③-④, bending and standing as transitional states were detected for around 10 frames.

**Figure 10:**
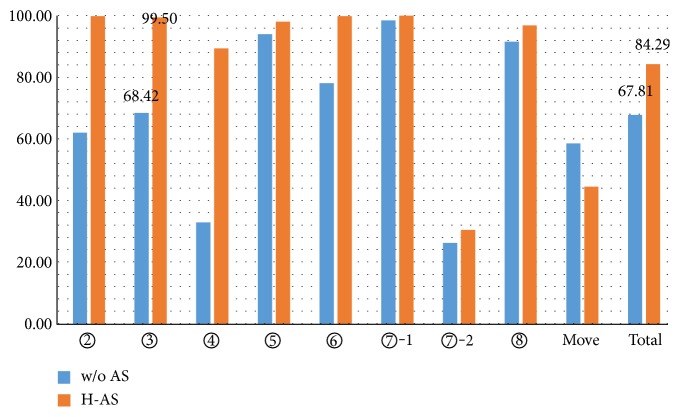
Accuracy (%) of behavior recognition of the w/o AS and H-AS. Note: the label “Move” means the walking for move behavior.

The comparison of recognition accuracy between the system with H-AS and the conventional system (no active sensing) is shown in Figure 10. Here, all the recorded frames and true frames of walking for move between sites (marked in italic in Table 5) were summed up. And the accuracy values shown are the average of that of two subjects. As shown in the graph, recognition accuracy of all the activities except walking for move was improved by applying active sensing.

In total, the accuracy was improved by H-AS (H-AS 84.29% versus w/o AS 67.81%). The accuracy of sitting and watching TV at ❸ was improved up to 99.50%, from 68.42% by the conventional system. This significant improvement could be the result of the viewpoint change of the robot during the experiments. For the activities except sitting and walking, improvement of recognition accuracy was confirmed, too, though, for those activities, irregularity was rarely detected. This is because that, taking the connection of sitting and standing as an example, the activities are sequentially conducted, a good observing viewpoint for sitting can be also good for the next subsequent activity, such as standing.

In Figure 10, it is also observed that the recognition accuracy of both walking for exercise at ❼ and walking for move was quite low. In fact, most frames of walking were misrecognized as standing, which may result from the low walking speed of the subject. In the activity recognition algorithm, the moving speed of subjects was also taken into consideration [11]. Basically, in the experiment, the subject walked slowly, since he was instructed to simulate the walking of the elderly. That is why the misrecognition occurred frequently. This can be further improved by adjusting behavior recognition algorithm.

Taking the accuracy values at different situations, and that of walking for move, of the 2 subjects as samples, a paired (w/o AS versus H-AS) *t*-test was performed. The *p* value is 0.0222, which means that there is significant difference between the two methods.

### 4.4. Experiment 3, Daily Living Activity Scenario 2

Table 6 shows a comparison between the accuracy at each simulation between the w/o AS, H-AS, and TM-AS. As shown in the table, at situation ②, accuracy by TM-AS reaches 88.2%. Comparing to 56.7% by using H-AS and 67.2% for w/o AS, this shows that TM-AS effectively improves the accuracy for the situation. The deep-back chair (as shown in the right part of Figure 7) is a different challenge from that of the wall and curtain, as tested in experiment 2. Depending on the observation angle, the deep back shades the upper body completely, without showing the two-peak feature, which is a decisive factor of the heuristics in H-AS. That is why the accuracy of H-AS is even worse than w/o AS.

On the other hand, at situation ④, which is also a sitting related situation, the accuracy of TM-AS is much higher than that of w/o AS. However, it is lower than that of H-AS, which is to be analyzed later in this subsection in detail. Sitting at both situations ② and ④ is analyzed. Table 6 shows the accuracy of both situations from all the three subjects, subject 1 (S1), subject 2 (S2), and subject 3 (S3). Performing *t*-test to the pairs of methods, as shown in the lower part of Table 7, there is a significant difference between the TM-AS and w/o AS and no significant difference between w/o AS and H-AS, or between H-AS and TM-AS.

**Figure 11:**
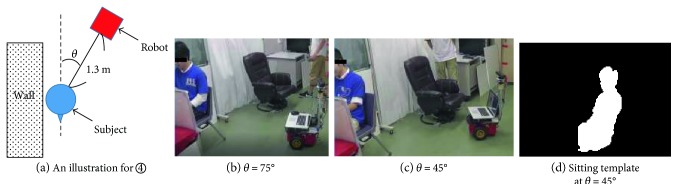
An explanation about the observation angle at situation ④.

The fact that the accuracy of TM-AS is lower than that of H-AS at the sitting situations in Table 7 can be explained as follows.  Figure 11(b) shows the illustration and photos for recognitions for situation ④. Here, the observation angle is defined as *θ* in Figure 11(a). At *θ* = 75° as shown in Figure 11(b), after adjusting the sensing range, the silhouette of the subject could be acquired and matched with one of the sitting templates prepared. A successful recognition was obtained when the robot observes the subject at an angle over 75°. However, not all the angles lead to a successful recognition.  Figure 11(c) shows the case when *θ* = 45°. Although by adjusting the sensing range, the silhouette of the sitting subject could be acquired clearly too, and it could not match any of the three sitting templates (front, left, and right as shown in Figure 2). Thus, the active sensing could not stop successfully within a time limit. It is reasonable to assume that an additional sitting template at around 45° will help to solve the problem.

In order to verify the aforementioned hypothesis, a 45° sitting template as shown in Figure 11(d) was added, and an additional supporting experiment at situation ④, with one subject sitting at the site, and the robot started from three observation angles, 30°, 45°, and 60°, was done. The results are tabulated in Table 8. As shown in the table, the accuracy at *θ* = 45° was improved for sitting for situation ④. Certainly, this raised a new question for the T-AS, that is, for each behavior, how many templates should be prepared for a recognition with good accuracy, which shall be investigated in near future.

**Figure 12:**
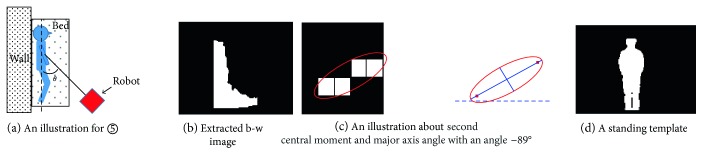
An explanation about misrecognition at situation ⑤.

**Figure 13:**
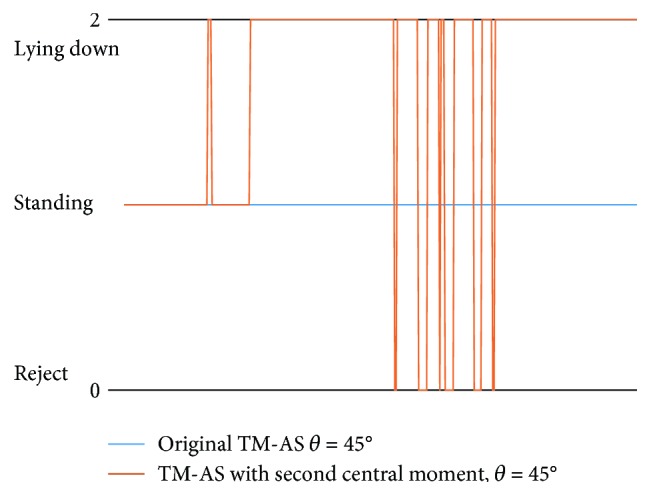
Time course of the recognized activity of the original TM-AS and an improved TM-AS.

Another result requiring further investigation is the unsatisfactory accuracy (46.2% for H-AS and 33.7% for TM-AS) at situation ⑤, as shown in Table 6 previously. After checking the results carefully, it is clear that, in most failed cases, the subject was lying in the bed and leaning against the wall, as shown in the illustration in Figure 12(a). Especially, when the angle between the robot and the subject *θ* is about 45°, this is more critical. Note, under the one-room setting of experiment 3, the distance among the furniture is small, and a 45° observation angle is more likely to happen than 90°. One example of the binary images extracted from the depth images of such cases is shown in Figure 12(b), in which, a portion of the wall (vertical part) and the lying human body (horizontal part) were merged. The upper part of this binary image is similar to the standing template (shown in Figure 12(d)). Therefore, the misrecognition of lying down on a bed as standing occurred.  Figure 13 shows the time course of the recognized activity of situation ⑤ of the original TM-AS (the blue line). The sitting behavior was misrecognized as standing by the original TM-AS.

This problem can be dealt with by introducing the second central moment [19] and making use of the orientation of the major axis of the binary image as shown in Figure 12(c). The orientation of the major axis (the long axis of the ellipse enclosing the binary image) can differentiate the pure standing and lying down with confusion from the wall. As an example, in Figure 12(d), the orientation of the major axis of a pure standing image is −89°.

In order to validate this idea, an additional supporting experiment was performed by one subject at situation ⑤. The cases with the observation angles 45° and 90° were tested.  Table 9 shows the results. The time course of the recognized activity by the improved TM-AS is shown by an orange line in Figure 13. Especially, the accuracy in the observation when angle was 45° was improved greatly from 0% to 64.75%, though there is still space for improvement. This will be further discussed in the next section.

## 5. Discussion

In this paper, an active sensing approach is proposed for a home monitoring robot where a categorization is introduced for further explorations for improving the accuracy of activity recognition with less time cost. It was targeted at uncertain situations where a subject under monitoring is indistinguishable with the surrounding objects in a real environment.

The performance of the active sensing depends greatly on the irregularity detection. H-AS is a method specifically designed for the targeted application. It is based on a heuristic for detecting a local irregular feature pattern, which depends on the activities to be recognized. Therefore, it can only categorize all the situations into two classes: regular and irregular. Through the experiments, it is found that the H-AS could deal with the ambiguous situations to a certain extent; however, when the subject was sitting very close to an object with similar sitting height as the subject, it failed to detect the irregularity. Moreover, the local irregular feature pattern could only cover a specific type of uncertain situations, thus ignore all the situations where changing the sensing parameters is needed. Despite that, the H-AS led to an improvement in the accuracy of activity recognition in a daily living activity scenario for those activities that happen frequently.

On the other hand, TM-AS realizes more general irregularity detection, in which activity-dependent templates representing normal situations were prepared for comparing with the current situations. The unmatched situations are judged as irregular for further sensing. Although TM-AS was only tested for the activity of sitting in two controlled scenarios in this study, in general cases, it could detect irregular situations against identified activity-dependent normal ones. In general, the template-matching-based irregularity detection is the nearest neighbor classifier that excludes the far data points as anomaly (irregularity). It is one of the canonical anomaly detection approaches. Other methods for anomaly detection, such as simple statistical methods and machine learning-based methods, including both the supervised learning (e.g., support vector machine) and unsupervised learning (e.g., different clustering methods), can be applied to the proposed framework. These can be further implemented and compared with the current approach in our future work.

In this study, the accuracy of recognizing the main behaviors of an elderly staying at home by a robot is examined. They are standing, walking, bending, sitting, lying down, and falling. The detection of behaviors and the change between two behaviors, such as from walking to sitting, are crucial for home monitoring for behavioral studies. The walking speed in this home monitoring scenario is set to be 1 km/hr–3 km/hr. Because of the low speed and the small change of speed, the study on walking was not further detailed into different walking speeds. Moreover, due to the low speed, it is hard to be distinguished from standing. Especially in experiment 2, at situation ⑦, the walking exercise required the subject to move forward and backward repeatedly and slowly, which makes the differentiation more difficult. Moreover, in this study, the activity recognized during the behavior changes was not counted, because the duration of behavior changes is much shorter than that of the behaviors during daily living, and real-time intervention is out of our scope. In the future, if the behavior changes need to be accurately monitored, for the purpose of, for example, real-time behavior support, then the influence of the speed on accuracy during behavior changes has to be investigated. In that case, the sampling rate and the computational efficiency of the system should be improved to meet the real-time requirement.

The aim of experiment 3 is to explore the limitation of the proposed AS algorithms. Based on the results, it is clearly shown that there are various kinds of confusing situations caused by a wall or by different types of furniture to different behaviors. These situations need to be identified by irregularity detection and further coped with by efficient active sensing. Experimentally, we have successfully shown the possibility of applying TM-AS to deal with the confusion caused at situations ④ and ⑤ in experiment 3. More systematic investigation is needed to verify the effectiveness of the improved TM-AS in a complete way. Especially, the following three issues shall be further investigated with immediate attention.

The first issue is that the accuracy of detection can considerably be affected by the number of available templates. More templates are needed for increasing the accuracy of detection. This was shown by the results and further investigation of situation ④ in experiment 3. Moreover, when the number of behaviors for detection increases, more templates are needed. Therefore, the templates for matching need to be generated carefully for expressing a complete set of normal situations as required. The clustering method [20] might be useful to generate the templates in an unsupervised way.

The second issue is that, as the environment turns to be more complex, the distance (similarity) index might be insufficient to differentiate the normal and irregular situations. This was shown by the results and further investigation of situation ⑤ in experiment 3, in which, an irregular situation of one behavior (e.g., lying down) was recognized as the normal situation of another behavior (e.g., standing). Therefore, multiple similarity indexes need to be applied to avoid the false-positive judgement. Furthermore, for some location-dependent critical confusing cases, the initial recognition could be taken as a hypothesis for active sensing to confirm. Each recognition result could be attached with a location-dependent or even behavior-dependent “belief” value, when the “belief” value is low, then an active sensing method could be initiated to confirm the first guess.

The third issue is that the efficiency of the exploration of sensing parameter shall be improved. For TM-AS, after an irregularity was identified based on a temporary activity, further explorations are encouraged by the method to explore the templates for all the activities for all the sensing parameters, which lowers its time efficiency. Therefore, for TM-AS, its time efficiency needs further improvement. This can be done by introducing a memory-based approach for the exploration of sensing parameter. If the robot could identify its situations, employ situation-dependent exploration strategy, and improve the strategy in an incremental way, more practical active sensing could be realized.

In this work, we aimed to realize the active sensing mechanism for robust sensing and behavior monitoring. In the near future, we will classify the Japanese home living environments into several types and do a set of experiments, with more subjects, to evaluate our monitoring mobile robot system in a comprehensive way.

## 6. Conclusion

This work is an advancement of the previously presented work based on the use of a mobile robot for monitoring the home-alone elderly. It addresses the problem of the uncertain situations, where the subject under monitoring is difficult to be distinguished from the background. A new approach is proposed where active sensing is applied for categorization of situations before further explorations for both detection accuracy improvement and cost minimization of time. For categorizing the situations, two schemes, H-AS and TM-AS, were employed for irregularity detections. Experiments were designed to compare and examine the performances of both active sensing schemes in terms of the detection accuracy and time cost for the targeted application. The proposed approach can detect ambiguities of surrounding objects and change the sensing parameters of the robot to eliminate the ambiguities. Experimental results show that significant improved accuracy of the system in both a controlled scenario and two home living scenarios has been achieved, with reasonable time cost.

For the proposed approach, remained issues are identified, analyzed, and discussed in detail. In our future work, the TM-AS is to be improved by well-prepared templates, hybridized similarity indexes, and optimized sensing parameter exploration strategy. Moreover, other common uncertain situations, such as occlusion by furniture, difficulties of localization and movement control due to uneven terrain, and frequent partial furniture layout alternations, will be resolved.

## Figures and Tables

**Pseudocode 1:**
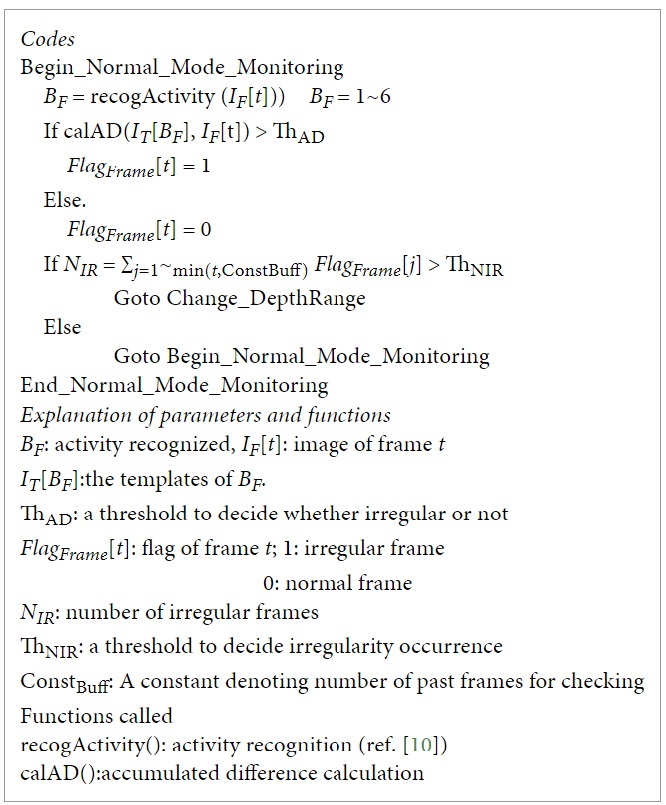
Pseudocode 1: Normal_Mode_Monitoring.

**Pseudocode 2:**
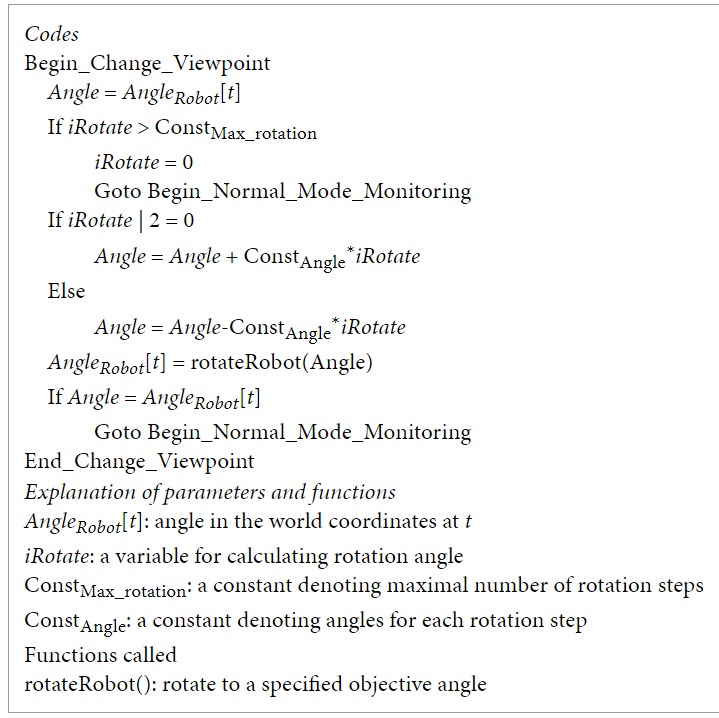
Pseudocode 2: Change_Viewpoint.

**Pseudocode 3:**
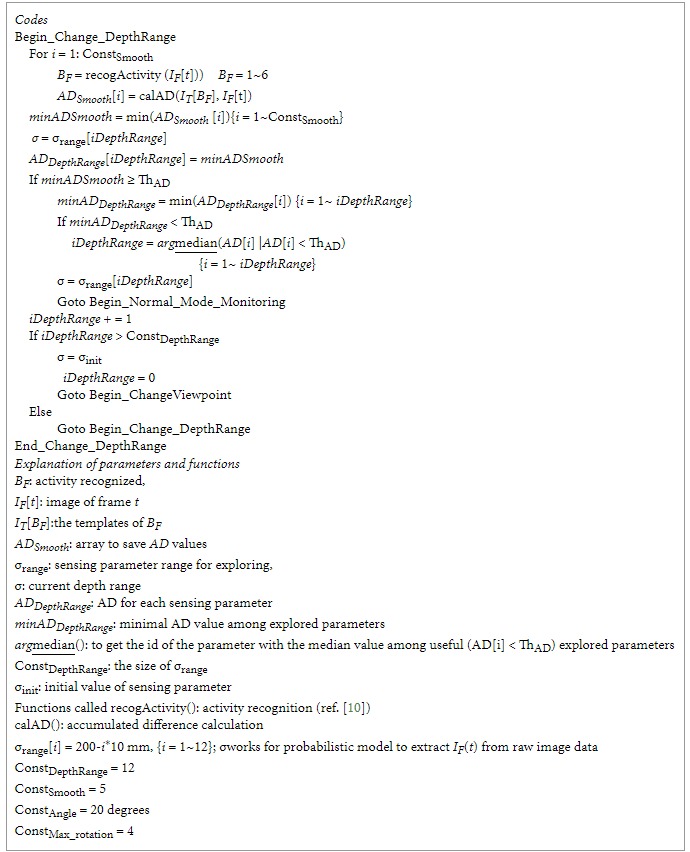
Pseudocode 3: Change_DepthRange.

**Table 1:** DB values of the accumulated difference (AD) for different features.

Feature	DB
AD_*d*_^upper^	0.558
AD_*d*_^lower^	0.520
AD_Anv_	0.490

**Table 2:** The accumulated difference (AD) of unit normal vector angle among the template images.

Template image	Template image								
	Standing (front)	Standing (side)	Sitting (front)	Sitting (left)	Sitting (right)	Bending (back)	Bending (left)	Bending (right)	Lying (down)
Standing (front)	0.0	20.5	17.6	42.3	41.6	25.9	63.4	62.6	52.4
Standing (side)	20.5	0.0	29.6	45.2	37.9	23.8	55.9	56.0	58.8
Sitting (front)	17.6	29.6	0.0	44.9	36.7	29.4	65.6	65.4	53.1
Sitting (left)	42.3	45.2	44.9	0.0	66.5	39.8	61.0	60.5	57.4
Sitting (right)	41.6	37.9	36.7	66.5	0.0	36.3	64.5	62.8	61.7
Bending (back)	25.9	23.8	29.4	39.8	36.3	0.0	45.0	43.0	51.9
Bending (left)	63.4	55.9	65.6	61.0	64.5	45.0	0.0	50.7	65.4
Bending (right)	62.6	56.0	65.4	60.5	62.8	43.0	50.7	0.0	70.9
Lying (down)	52.4	58.8	53.1	57.4	61.7	51.9	65.4	70.9	0.0

**Table 3:** The activity recognition accuracy for sitting position for the three areas.

		Area ①	Area ②	Area ③
w/o AS (conventional)	Average frame number	292	259	217
	Recognition accuracy (%)	100.0	16.8	0.0

H-AS	Average frame number	282	201	265
	Recognition accuracy (%)	100.0	79.6	1.4

TM-AS	Average frame number	247	250	237
	Recognition accuracy (%)	100.0	84.7	52.9

**Table 4:** The time cost of AS (unit: second), from the first irregularity detected to the last sensing parameter change.

	Area ②		Area ③
AS trial number	Heuristic-based AS	Template-matching-based AS	Template-matching-based AS
Trial 1	14.5	10.9	27.3
Trial 2	10.4	13.9	29.9
Trial 3	7.74	13.9	11.9

**Table 5:** The accuracy of behavior recognition of subject 1, w/o active Sensing.

Situation number	Behavior	Video frames	True frames	Accuracy (%)	Situation number	Behavior	Video frames	True frames	Accuracy (%)
①-②	*Walking*	81	52	64.20	⑤	Sitting newspaper	4514	4292	95.08
②	Bending (washing)	177	164	92.66	⑤-⑥	*Walking*	124	75	60.48
②-③	*Walking*	61	43	70.49	⑥	Sitting book	5564	5075	91.21
③	Sitting (TV)	4711	1795	38.10	⑥-⑦	*Walking*	128	79	61.72
③-④	*Walking*	101	29	28.71	⑦-1	**Standing**	**385**	**385**	**100.00**
					⑦-2	**Walking**	**1164**	**477**	**40.98**
④	Bending (refrigerator)	266	175	65.79	⑦-⑧	*Walking*	292	147	50.34
④-⑤	*Walking*	28	17	60.71	⑧	Lying down	3371	2889	85.70

**Table 6:** Comparison of accuracy at each situation between w/o AS, H-AS, and TM-AS^∗^.

	w/o AS				H-AS				TM-AS			
Situation ID	②	③	④	⑤	②	③	④	⑤	②	③	④	⑤
Video frame	4389	4413	4149	3248	4402	4718	4446	4540	4640	4809	4614	4185
True Frame	2950	4364	571	691	2494	4366	4089	2096	4102	4791	3524	1412
Accuracy (%)	67.2	98.9	13.8	21.3	56.7	92.5	92.0	46.2	**88.4**	99.6	76.4	33.7

^∗^Note: the numbers of recorded video frame and true frame are the average of three subjects

**Table 7:** Comparison of accuracy of three subjects at situations ② and ④.

Subject ID-situation	w/o AS	H-AS	TM-AS
S1-②	64.6	96.5	90.6
S1-④	36.3	89.8	96.6
S2-②	68.9	13.0	88.6
S2-④	0.0	95.2	76.4
S3-②	68.1	52.7	85.9
S3-④	0.0	91.8	54.7
w/o AS versus H-AS	*t*-test: *p* = 0.113, no significant difference		
w/o AS versus T-AS	*t*-test: *p* = 0.017, with significant difference		
H-AS versus T-AS	*t*-test: *p* = 0.564, no significant difference		

**Table 8:** Comparison between the w/o and with a new 45° sitting template, for different initial observation angles (30°, 45°, and 60°).

	w/o the 45° template			With the 45° template		
Observation angle	30°	45°	60°	30°	45°	60°
Video frame	400	400	400	400	400	400
True frame	330	337	362	358	362	367
Accuracy (%)	82.5	84.25	90.50	89.50	90.50	91.75

**Table 9:** The accuracy of the original TM-AS and an improved TM-AS at situation ⑤.

	w/o the major axis angle		With the major axis angle	
Observation angle	45°	90°	45°	90°
Video frame	400	400	400	400
True frame	0	332	259	333
Accuracy (%)	0	83	64.75	83.25

## Appendix

Consider Pseudocodes 1, 2, and 3 for exploring sensing parameters with irregularity detection based on template matching.
